# Discrepancies in Outcome Reporting Exist Between Protocols and Published Oral Health Cochrane Systematic Reviews

**DOI:** 10.1371/journal.pone.0137667

**Published:** 2015-09-14

**Authors:** Nikolaos Pandis, Padhraig S. Fleming, Helen Worthington, Kerry Dwan, Georgia Salanti

**Affiliations:** 1 Department of Hygiene and Epidemiology, University of Ioannina School of Medicine, Ioannina, Greece; 2 Department of Orthodontics and Dentofacial Orthopedics, Dental School/Medical Faculty, University of Bern, Bern, Switzerland; 3 Barts and The London School of Medicine and Dentistry, Queen Mary University of London, London, United Kingdom; 4 Cochrane Oral Health Group, School of Dentistry, The University of Manchester, Manchester, United Kingdom; 5 Institute of Translational Medicine, Department of Biostatistics, University of Liverpool, Liverpool, United Kingdom; University of Florence, ITALY

## Abstract

**Objectives:**

To assess discrepancies in the analyzed outcomes between protocols and published reviews within Cochrane oral health systematic reviews (COHG) on the Cochrane Database of Systematic Reviews (CDSR).

**Study Design and Setting:**

All COHG systematic reviews on the CDSR and the corresponding protocols were retrieved in November 2014 and information on the reported outcomes was recorded. Data was collected at the systematic review level by two reviewers independently.

**Results:**

One hundred and fifty two reviews were included. In relation to primary outcomes, 11.2% were downgraded to secondary outcomes, 9.9% were omitted altogether in the final publication and new primary outcomes were identified in 18.4% of publications. For secondary outcomes, 2% were upgraded to primary, 12.5% were omitted and 30.9% were newly introduced in the publication. Overall, 45.4% of reviews had at least one discrepancy when compared to the protocol; these were reported in 14.5% reviews. The number of review updates appears to be associated with discrepancies between final review and protocol (OR: 3.18, 95% CI: 1.77, 5.74, p<0.001). The risk of reporting significant results was lower for both downgraded outcomes [RR: 0.52, 95% CI: 0.17, 1.58, p = 0.24] and upgraded or newly introduced outcomes [RR: 0.77, 95% CI: 0.36, 1.64, p = 0.50] compared to outcomes with no discrepancies. The risk of reporting significant results was higher for upgraded or newly introduced outcomes compared to downgraded outcomes (RR = 1.19, 95% CI: 0.65, 2.16, p = 0.57). None of the comparisons reached statistical significance.

**Conclusion:**

While no evidence of selective outcome reporting was found in this study, based on the present analysis of SRs published within COHG systematic reviews, discrepancies between outcomes in pre-published protocols and final reviews continue to be common. Solutions such as the use of standardized outcomes to reduce the prevalence of this issue may need to be explored.

## Introduction

Systematic reviews (SRs) for interventions provide the basis for evaluating the body of evidence and by extension are uniquely influential in leading to healthcare recommendations [1,2]. However, inappropriate practices such as selective inclusion of trials, selective reporting of outcomes and results-driven reporting can bias estimates of treatment effects culminating in compromised patient care, healthcare decisions and service configuration [3,4]. Selective outcome reporting involving preferential reporting of specific data or outcomes within a study is a recognized problem and has been investigated in respect of randomized controlled trials (RCTs), in particular [5–9].

While research on selective reporting has focused both on RCT s and non-randomized studies [10,11], similar issues may arise within SRs [12]. At the systematic review level, selective reporting may develop for a variety of reasons, for example, due to the use of multiple measurement scales, outcomes or time points [13,14] and selective inclusion of specific outcomes. Arbitrary inclusion of outcomes based on *post hoc* results-driven decisions may bias the conclusions from subsequent syntheses [13,15].

A recent systematic review [15] has highlighted among other issues the paucity of information on selective reporting based on comparisons between review protocols and final systematic review reports. The limited number of meta-epidemiological analyses identified were restricted to cystic fibrosis reviews on the Cochrane Database of Systematic Reviews (CDSR) prior to 2010 [16], or involved analysis of selected issues within the CDSR over three time periods between 2000 and 2008 [17–19].

Historically, with the notable exception of the CDSR, systematic reviews have lacked pre-published protocols. This has changed following the relatively recent introduction of the PROSPERO database, and publication of SR protocols in isolation in a dedicated journal (www.systematicreviewsjournal.com)[20], with reporting on the existence of a pre-published protocol also encouraged within the PRISMA guidelines [2]. To our knowledge, while the existence and impact of outcome reporting bias within dental SRs has been acknowledged [21], there are no reports addressing selective reporting and discrepancies between systematic review protocols and final reports within the oral health field. Therefore, the aim of the present study was to explore the prevalence and nature of selective reporting and factors associated with selective reporting within SRs published by the Cochrane Oral Health Group (COHG) in the CDSR by analyzing discrepancies between systematic review protocols and final reports.

## Methods

All systematic reviews in the Cochrane Oral Health Group (COHG) published in the CDSR until November, 2014 were screened for final reports and pre-published protocols. The first author (NP) contacted the COHG in order to retrieve review protocols that were not available in the Cochrane library. Reviews where the protocol could not be found as well as those reviews published in duplicate were to be excluded from further analysis.

Data were independently extracted and entered on pre-piloted standardized forms for the eligible studies. Initial calibration was performed between the two researchers (NP, PSF) on 10 articles. Disagreements were resolved by discussion or, if necessary, with adjudication by a third reviewer (KD). Information obtained included the number and type of primary and secondary outcomes both within the protocol and in the final publication. If a review did not distinguish between primary and secondary outcomes [unlabelled outcomes], the first three outcomes listed were taken to be the primary outcomes and the rest considered as secondary outcomes. Specific details as to whether outcomes present in the protocol were omitted, upgraded or downgraded as well as inclusion of outcomes in the final report that were absent from the protocol were recorded. Data relating to year of publication of the protocol and the final report, and any previous versions of the review, number of authors, geographical location of the corresponding author, whether the collaboration involved a single or multiple centers and subject area were also extracted. In addition, information concerning statistical significance was extracted for one primary outcome from the reviews with applicable meta-analyses. The primary review comparison was assumed for each review[19] according to the following hierarchy by selecting that which met the first of the following criteria: (1) an intervention comparison described in the protocol as the primary review comparison; (2) the first intervention comparison mentioned in the objectives of the protocol; (3) an intervention comparison described in the review as the primary review comparison; (4) the first intervention comparison mentioned in the objectives of the review; (5) the intervention comparison used in the first meta-analysis presented in the review.

## Data Analysis

Descriptive statistics were calculated for all included articles related to the following variables: geographical representation, number of research centers, number of review updates, number of authors and year of publication. The year of publication was converted to a binary variable [<2008, ≥ 2008, as from 2008 Cochrane included a requirement to declare changes between protocol and review]. Cross-tabulations were undertaken to investigate associations between the presence or absence of discrepancies and review characteristics. A logistic model was also fitted in order to assess and quantify any association of discrepancies between protocol and final review and review characteristics. Finally, upgrades, downgrades, new introductions and omissions between protocol entries and final reports for all outcomes listed were tabulated. Risk ratios were calculated in order to examine the relationship between possible discrepancies in the primary outcomes and statistical significance. All statistical analyses were conducted with Stata^®^ version 13.1 software (Stata Corporation, College Station, Texas, USA).

## Results

**Fig 1:**
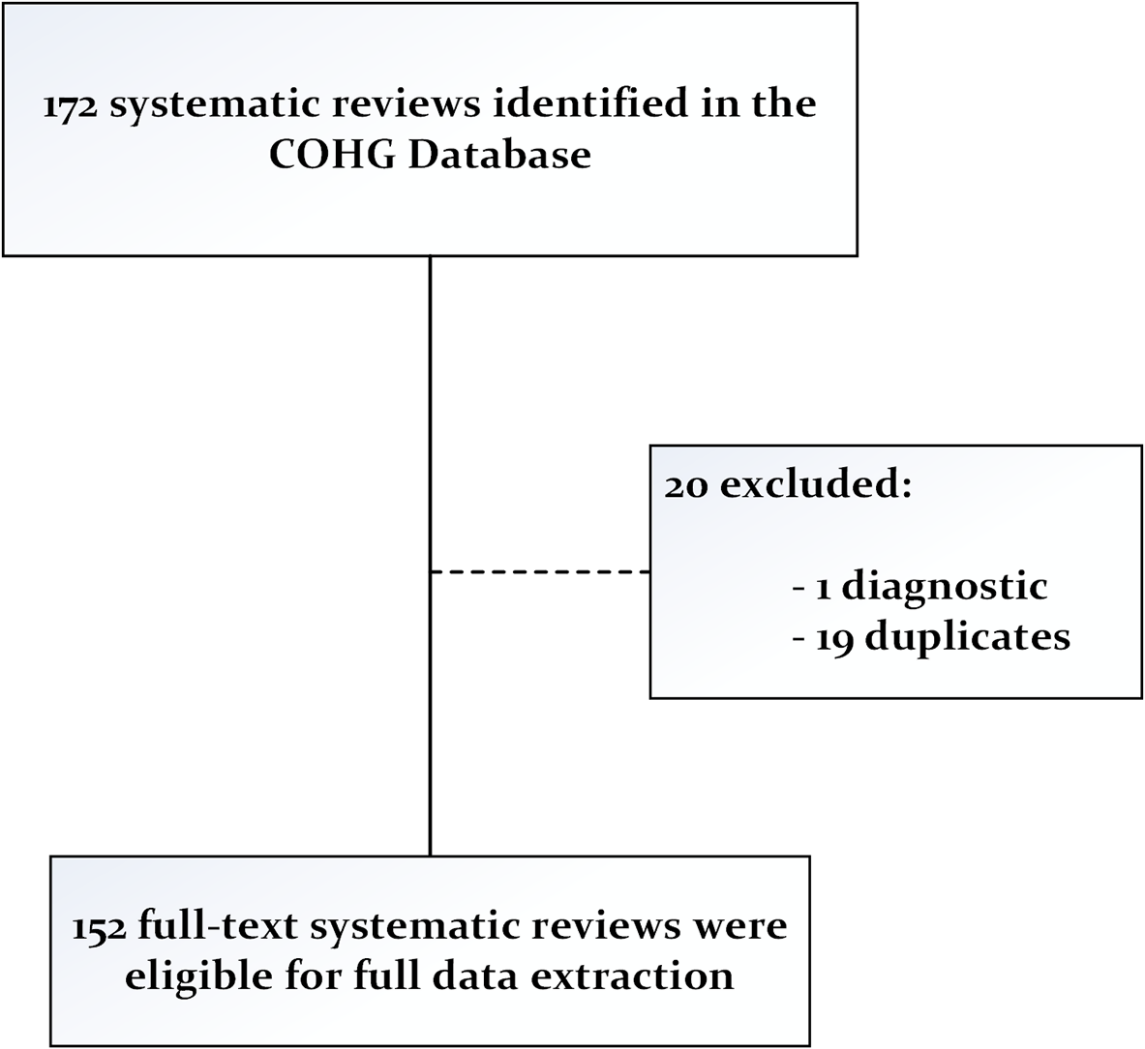
SR selection flow diagram.

Initially 172 titles were identified in the COHG database of which 19 reviews were excluded as duplicates [same title and DOI] and one diagnostic review was excluded (Fig 1). From the remaining 152 reviews 116 (76.3%) had accessible protocols next to the review under the ‘‘Protocol and previous versions” section on the Cochrane Library. The COHG provided the protocols for 36 reviews for which the protocols were not available in the Cochrane library.

A variety of conditions, interventions and outcomes were considered with the highest number of reviews dealing with dental caries, orthodontics, periodontics and implantology (S1 Table). The largest proportion of reviews originated in the UK according to the details of the corresponding authors (S2 Table).

The median number of labelled primary outcomes was 2 (range: 0–11) and secondary outcomes was 4 (range: 0–36) both in the protocols and published reviews. Overall, 51 protocols (33.6%) and 34 final reviews (22.4%) did not distinguish between primary and secondary outcomes. This was less common among reviews published after 2008 compared to those published prior to 2008 (OR: 0.33, 95% CI: 0.14, 0.75, p = 0.01) but no important improvement was observed at protocol level for outcome distinction after 2008 compared to the previous period (OR: 0.54, 95% CI: 0.25, 1.16, p = 0.11). The distribution of individual discrepancies between protocols and reviews per geographical area, single or multicenter collaboration, number of review updates, number of authors and publication period is presented in Table 1.

**Table 1:** Distribution of any discrepancies between protocols and reviews per geographical area, single or multicenter collaboration, number of review updates, number of authors and review publication period.

		Discrepancy				p-value
**Continent of origin of corresponding author**	Total	no	no	yes	yes	
	No.	No.	%	No.	%	
Middle East & Africa	10	7	70	3	30	0.09*
Americas	15	12	80	3	20	
Asia	11	7	64	4	36	
Europe	116	57	49	59	51	
	152	79	52	73	48	
**Collaboration between centers**						
No	4	3	75	1	25	0.41*
Yes	148	80	54	68	48	
**Number of review updates**						
No updates	104	69	66	35	34	<0.001**
1 update	29	12	41	17	59	
≥2 updates	19	2	11	17	89	
**Number of authors**						
2–3	21	11	52	10	48	0.98**
4–5	73	40	55	33	45	
≥ 6	58	32	55	26	45	
**Review publication period**						
<2008	36	24	67	12	33	0.10**
≥2008	116	59	51	57	49	
**Total**	152	83	55	69	45	

*Fisher’s exact test

** Chi2 test

According to the multivariable logistic model (Table 2) the number of review updates appears to be associated with discrepancies between final review and protocol (OR: 3.18, 95% CI: 1.77, 5.74, p<0.001). Geographical area, single or multicenter collaboration, review publication year and number of authors do not appear to be important predictors of discrepancies between protocols and final reports.

**Table 2:** Adjusted odds ratios and associated 95% confidence intervals for the association between any discrepancies (protocol vs final report) and review characteristics.

Characteristic	OR	95% CI	p-value
Number of authors [per unit]	1.10	0.90, 1.34	0.34
Geographical region			
Americas	reference	-	-
Middle East & Africa	1.95	0.29, 13.34	0.50
Asia	2.15	0.35, 13.33	0.41
Europe	3.09	0.78, 12.29	0.11
Number of updates [per unit]	3.18	1.77, 5.74	<0.001
Review publication year			
<2008	reference		
= >2008	1.33	0.54, 3.29	0.54
Collaboration between centers			
No	reference	-	-
Yes	2.55	0.16, 41.65	0.51

The detailed analysis of discrepancies (Table 3) revealed that the primary outcome was not stated in 3.9% (6/152) of protocols but stated in all final reports. In 30.1% (49/152) of the reviews the primary outcomes were not consistent in the protocol and the report while secondary outcomes were not the same in 43.4% of the reviews (66/152). In 11.2% (17/152) of the reviews the primary outcomes were downgraded to secondary, in 9.9% of the reviews (15/152) they were omitted and in 18.4% (28/152) new primary outcomes were introduced. In four reviews out of 152 (2.6%) a change in the definition was identified. In 2% (3/152) of the reviews secondary outcomes were upgraded to primary, 12.5% (19/152) were omitted and in 30.9% (47/152) new secondary outcomes were introduced. No outcome definition change was identified for secondary outcomes. Overall, 45.4% (69/152) of the reviews presented at least one discrepancy but only in 14.5% (10/69), all published after 2008, were discrepancies reported in the text (S3 Table). It is important to point out that not all declared changes were explained in detail or justified.

**Table 3:** Tabulation of type of discrepancy between protocols and reviews for primary and secondary outcomes at the review level.

Type of discrepancy		
	n	%
**Primary outcome variable not stated in the protocol**	6/152	3.9
**Primary outcome variable not stated in the published report**	0/152	0.0
**All primary outcome(s) stated in the protocol is/are not the same as in the published report**	47/152	30.9
**One or several primary outcome(s) stated in the protocol is downgraded to secondary in the published report**	17/152	11.2
**One or several primary outcome stated in the protocol is/are omitted from the published report**	15/152	9.9
**One or several new primary outcome(s) that was/were not stated in the protocol is included in the published report**	28/152	18.4
**The definition of one or several primary outcome(s) was different in the protocol compared to the published report**	4/152	2.6
**All secondary outcomes stated in the protocol are not the same as in the published report**	66/152	43.4
**One or several non-primary outcome(s) in the protocol is/are changed to primary in the published report**	3/152	2.0
**One or several secondary outcome(s) stated in the protocol is/are omitted from the published**	19/152	12.5
**One or several new secondary outcome(s) that was/were not stated in the protocol is/are included in the published report**	47/152	30.9
**The definition of one or several secondary outcome(s) was different in the protocol compared to the published report**	0/152	0.0

Of the 152 reviews assessed, 89 did not have a meta-analysis or the meta-analysis did not relate to the primary outcomes. Within the remaining reviews the distribution of discrepancies for the primary outcome by statistical significance is shown in Table 4. The risk of reporting significant results was lower for downgraded outcomes [RR: 0.52, 95% CI: 0.17, 1.58, p = 0.24] and upgraded or newly introduced outcomes [RR: 0.77, 95% CI: 0.36, 1.64, p = 0.50]. The risk of a significant result was higher for upgraded/new outcomes (RR = 1.19, 95% CI: 0.65, 2.16, p = 0.57) compared to outcome downgrades. None of the comparisons reached statistical significance.

**Table 4:** Tabulation of counts, risk ratios and associated 95% confidence intervals and p-values per type of discrepancy and statistical significance for primary outcomes only.

	**Total**	**Significant (<0.05)**	**Non-significant (>0.05)**	**Risk Ratio (95% CI)**	**p-value**
**No discrepancy**	24	16	8	Reference	-
**Downgrade**	9	4	5	0.52 (0.17, 1.58)	0.24
**Upgrade/Inclusion**	16	9	7	0.77 (0.36, 1.64)	0.50
	**Total**	**Significant (<0.05)**	**Non-significant (>0.05)**	**Risk Ratio (95% CI)**	**p-value**
**Downgrade**	9	4	5	Reference	-
**Upgrade/Inclusion**	16	9	7	1.19 (0.65, 2.16)	0.57

## Discussion

### Main findings

This meta-epidemiological study attempted to identify discrepancies between 152 COHG systematic reviews and protocols in order to shed some light on selective reporting in oral health systematic reviews. The reviews covered a wide range of topics and were produced by authors located predominantly in the UK. Similar reviews have been undertaken within Cystic Fibrosis reviews on the CDSR [16] or generally within specific issues of the CDSR covering a range of review groups [17–19]. The most recent of the latter reviews focused on a period up to 2010 [16] identifying a discrepancy rate of up to 39%, while an earlier review alluded to a discrepancy rate of 22% [17]. Our findings spanning a 16-year period indicate that within SRs in the COHG discrepancies continue to be highly prevalent, with a prevalence of 45% for primary outcomes. The risk for discrepancy increased with increasing number of review updates. This is intuitive as additional outcomes can become more or less important over time with, for example, increasing emphasis on important outcomes in recent years. Moreover, new reviewers may differ from previous reviewers in relation to the priority with which they assign to certain outcomes. The risk of statistically significant results was lower for both outcome downgrades and upgrades/new inclusion compared to outcomes with no discrepancy. The finding for upgrades/new inclusions were opposite to what was expected. The risk of statistically significant results, consistent with other studies, was higher for outcome upgrades/new inclusions compared to outcome downgrades. None of those finding reach statistical significance however, as data was thin.

Some of these problems with discrepancies between protocols and reviews will be addressed when the COHG completes research being undertaken on Core Outcome Measures in Effectiveness Trials (COMET; http://www.comet-initiative.org) which will provide a list of core outcomes to be included in reviews being undertaken in certain areas of oral health.

### Limitations and strengths

A large number of reviews were captured relative to published previous assessments, which have ranged from 46 to 288 SRs [16,17]. Articles were identified by just one author in the present study; however, as just a single database was searched with articles readily accessible, the risk of selection bias and inappropriate omission of relevant articles is very low. We are, therefore, confident that the present findings are a true reflection of the discrepancy rate both for primary and secondary outcomes with the Oral Health Group of the CDSR. The Oral Health Group relates to reviews published within the dentistry and oral and maxillofacial surgery; previous meta-epidemiological assessments have confirmed that dental SRs are equally susceptible to methodological and reporting weaknesses as other biomedical areas [22–25]. It is, therefore, likely that the findings from the present review are representative of the CDSR more broadly.

### Findings in context

A large number of discrepancies were identified between protocols and final reports for both primary and secondary outcomes; these included introduction of new outcomes, upgrades, downgrades, omissions and definition changes. Since 2008, the Cochrane Collaboration requires that published reviews disclose discrepancies between protocols and reports. In this study a low percentage (14.5%) of outcome discrepancies were declared. This figure is in keeping with similar research with Kirkham *et al*. reporting acknowledgment of *post hoc* changes in just 6% of 64 reviews found to have discrepancies [17]. However, in a more recent review [16] discrepancies were mentioned in 39%, albeit based on a smaller sample of 46 reviews overall. These figures, however, may indicate a lack of awareness among review authors and failure of reviewers to cross-reference final submissions with pre-published protocols. While there is ample evidence that SRs published on the CDSR are of higher methodological quality than those published elsewhere [26–28] the stipulation of disclosure of discrepancies within the CDSR does not appear to have had the desired effect either within the COHG or more generally.

In the present subset, no statistically significant association between alteration in outcomes (upgrade from secondary to primary, introduction of new outcomes, downgrades) and statistically significant of the meta-analysis results was found. The risk for reporting significant results was higher for upgraded or newly introduced outcomes compared to downgraded outcomes, although a non-statistically significant finding. Kirkham *et al*. [19] found an increased risk of a significant result with discrepancies including new inclusion or upgrades but not with downgrades. The discordance in relation to upgrades/new inclusion of outcomes between these studies may be attributed to the relatively small number of included meta-analyses in the present study but may also relate to the type of relevant outcomes.

### Implications of results

The continued stubbornly high outcome discrepancy rate within the CDSR suggests that the prerequisite of delineating changes between protocol and submitted review is ineffective. However, external screening by the Cochrane Editorial Unit from 2013 has helped to ensure these changes are documented and justified in new reviews published since September 2013.

An alternative may be to consider a streamlined approach incorporating checks ensuring that outcomes in the review correspond with those delineated in the protocols. This approach could be implemented at the editorial level with proprietary software. For example, outcomes from the protocol may be automatically transferred to the review. Where authors wish to make changes in the review those changes would only be permitted if a declaration for changes field was completed. The editor could then be alerted of the outcome changes between protocol and review prompting the need for approval and registering the change. In order to improve transparency consideration could be given to publication of formal protocol amendments within the CDSR, although this would require external approval and may lead to a more protracted publication process.

The importance of setting core outcomes common to individual trials and reviews in order to enhance usability and transparency of reports and to reduce variability in outcome specification [29] can also be considered as an important step in reducing discrepancies [30,31]. However, the introduction of standardised core outcome sets may, initially at least, result in more discrepancies between reviews and protocols (particularly in review updates) as review authors adjust their outcomes to reflect consensus core outcome sets. In time, however, it is likely that as core outcome sets become more established it is likely that the prevalence of selective reporting and outcome alterations would diminish.

In terms of predictors, it appears differences between protocols and published reviews were more likely with increasing numbers of review updates. This finding is intuitive and may reflect the fact that reviews may span periods in excess of a decade and may, therefore, involve different author groups. Moreover, there are instances of multiple reviews being derived from the same protocol. The correct protocols for these “split” reviews were provided by the COHG group office and were clearly identified. It may, however, be sensible to consider more regular update of SR protocols within the CDSR after a defined time period to mitigate this.

In conclusion, the requirement to declare outcome changes between protocols and reviews which was implemented in 2008 is certainly a positive, there is further evidence that discrepancies still exist based on this analysis of SRs published within the Oral Health Group since its establishment. Alternative approaches to reduce the prevalence of this issue including the use of Core Outcome Sets need to be explored.

## Supporting Information

S1 File Dataset. (XLSX)

S1 Table Distribution of discrepancies between protocols and reviews per subject area. (DOCX)

S2 Table Distribution of discrepancies between protocols and reviews per country of origin. (DOCX)

S3 Table Explanation provided by authors for outcome changes between protocol and review. (DOCX)
