# Supplementary material for: Discrepancies in Outcome Reporting Exist Between Protocols and Published Oral Health Cochrane Systematic Reviews
Source: PLoS One. 2015 Sep 14;10(9):e0137667. doi: 10.1371/journal.pone.0137667 (PMC4569349; doi:10.1371/journal.pone.0137667)
Supplement: S1 Table — (DOCX) [file pone.0137667.s002.docx]

S1 Table. Distribution of discrepancies between protocols and reviews per subject area.

| Classification |  | **Discrepancy** | | | |
| --- | --- | --- | --- | --- | --- |
|  | Total | NO | NO* | YES | YES* |
|  | No. | No. | % | No. | % |
| Anesthesia | 1 | 1 | 100 | 0 | 0 |
| Antibiotics | 4 | 2 | 50 | 2 | 50 |
| Burning mouth syndrome | 1 | 1 | 100 | 0 | 0 |
| Cancer prevention | 1 | 0 | 0 | 1 | 100 |
| Cancer treatment | 4 | 1 | 25 | 3 | 75 |
| Central giant cell granuloma | 1 | 1 | 100 | 0 | 0 |
| Cleft lip & palate | 3 | 1 | 33 | 2 | 67 |
| Complications of surgery | 2 | 0 | 0 | 2 | 100 |
| Cosmetic therapy | 1 | 1 | 100 | 0 | 0 |
| Dental anxiety | 2 | 1 | 50 | 1 | 50 |
| Dental caries prevention | 19 | 14 | 74 | 5 | 26 |
| Dental caries treatment | 20 | 12 | 60 | 8 | 40 |
| Dentistry practice and systems | 1 | 1 | 100 | 0 | 0 |
| Dry mouth | 1 | 1 | 100 | 0 | 0 |
| Gingivostomatitis | 1 | 1 | 100 | 0 | 0 |
| Halitosis | 2 | 2 | 100 | 0 | 0 |
| Impacted teeth | 1 | 1 | 100 | 0 | 0 |
| Implants and prosthesis | 16 | 7 | 44 | 9 | 56 |
| Maintenance | 1 | 1 | 100 | 0 | 0 |
| Oral care for cancer patients | 5 | 0 | 0 | 5 | 100 |
| Oral hygiene | 3 | 2 | 67 | 1 | 33 |
| Oral lesions | 2 | 2 | 100 | 0 | 0 |
| Oral leukoplakia | 1 | 0 | 0 | 1 | 100 |
| Oral lichen planus | 2 | 0 | 0 | 2 | 100 |
| Oral mucosotis | 1 | 0 | 0 | 1 | 100 |
| Oral pain | 9 | 5 | 56 | 4 | 44 |
| Oral submucous fibrosis | 1 | 1 | 100 | 0 | 0 |
| Oral ulcers | 2 | 1 | 50 | 1 | 50 |
| Orthodontic treatment | 24 | 13 | 54 | 11 | 46 |
| Periodontal disease associated | 2 | 2 | 100 | 0 | 0 |
| Periodontal disease prevention | 3 | 0 | 0 | 3 | 100 |
| Periodontal disease treatment | 8 | 4 | 50 | 4 | 50 |
| Removal of third molars | 2 | 0 | 0 | 2 | 100 |
| Traumatic injury | 5 | 4 | 80 | 1 | 20 |
| Total | 152 | 83 | 55 | 69 | 45 |

* Row percentages
