# Supplementary material for: Discrepancies in Outcome Reporting Exist Between Protocols and Published Oral Health Cochrane Systematic Reviews
Source: PLoS One. 2015 Sep 14;10(9):e0137667. doi: 10.1371/journal.pone.0137667 (PMC4569349; doi:10.1371/journal.pone.0137667)
Supplement: S2 Table — (DOCX) [file pone.0137667.s003.docx]

S2 Table. Distribution of discrepancies between protocols and reviews per country of origin

| **Country of origin of corresponding author** |  | **discrepancy** | | | |
| --- | --- | --- | --- | --- | --- |
|  | Total | NO | NO* | YES | YES* |
|  | No. | No. | % | No. | % |
| Argentina | 1 | 1 | 100 | 0 | 0 |
| Bahrain | 6 | 4 | 67 | 2 | 33 |
| Brazil | 12 | 10 | 83 | 2 | 17 |
| China | 7 | 4 | 57 | 3 | 43 |
| Croatia | 1 | 1 | 100 | 0 | 0 |
| Denmark | 1 | 1 | 100 | 0 | 0 |
| Finland | 2 | 1 | 50 | 1 | 50 |
| France | 3 | 2 | 67 | 1 | 33 |
| Germany | 2 | 2 | 100 | 0 | 0 |
| Holland | 1 | 0 | 0 | 1 | 100 |
| Iran | 1 | 1 | 100 | 0 | 0 |
| Ireland | 3 | 2 | 67 | 1 | 33 |
| Italy | 6 | 2 | 33 | 4 | 67 |
| Japan | 1 | 1 | 100 | 0 | 0 |
| Mexico | 1 | 1 | 100 | 0 | 0 |
| Nigeria | 1 | 1 | 100 | 0 | 0 |
| Oman | 1 | 1 | 100 | 0 | 0 |
| Singapore | 1 | 1 | 100 | 0 | 0 |
| South Africa | 1 | 0 | 0 | 1 | 100 |
| Switzerland | 1 | 0 | 0 | 1 | 100 |
| Syria | 1 | 1 | 100 | 0 | 0 |
| Thailand | 1 | 0 | 0 | 1 | 100 |
| UK | 95 | 45 | 47 | 50 | 53 |
| USA | 2 | 1 | 50 | 1 | 50 |
| Total | 152 | 83 | 55 | 69 | 45 |

* Row percentages
