# Supplementary material for: Discrepancies in Outcome Reporting Exist Between Protocols and Published Oral Health Cochrane Systematic Reviews
Source: PLoS One. 2015 Sep 14;10(9):e0137667. doi: 10.1371/journal.pone.0137667 (PMC4569349; doi:10.1371/journal.pone.0137667)
Supplement: S3 Table — (DOCX) [file pone.0137667.s004.docx]

S3 Table. Explanation provided by authors for outcome changes between protocol and review.

| **Author** | **Explanation provided** |
| --- | --- |
|  |  |
| Guo[1] | We have revised the primary and secondary outcomes to make them more precise |
| Yengopal [2] | Other clinically important outcomes (e.g. gingival health and occlusion) have been added as secondary outcomes. Some included papers provided information on outcomes deemed to be clinically important to oral health professionals by the authors of these trials.  These outcomes (e.g. gingival health and occlusion) did not appear in the original protocol and were added to provide a wider spectrum of outcomes that may be considered as useful to clinicians and consumers. |
| Rasines Alcaraz [3] | Only data on permanent posterior teeth were reported in this review  In the protocol, survival rate was listed as the primary outcome but the review lists failure rate as primary outcome. Failure rate is reported in this review as a proxy for survival rate |
| Coulthart [4] | The primary outcome of ’altered sensation’ in the protocol has been changed to ’patient-reported altered sensation’ for the review |
| Daly [5] | We added the primary outcome for treatment of dry socket: time to heal. |
|  |  |
| Coulthart [6] | There have been some changes to the prespecified outcomes and prioritisation of outcomes |
| Furness[7] | The protocol for this review stated that quality of life would be a primary outcome for this review. Quality of life is an important outcome, for both patients with oral cavity and oropharyngeal cancers and their doctors. In this deadly and disfiguring disease, searching for treatments that offer an improvement in both quantity and quality of life for patients motivates the large body of research into the management of this disease. The search for effective chemotherapies is motivated at least in part by the desire to avoid patients having to undergo radical disfiguring surgery with resultant loss of function.  However, as the review has progressed we have found the large quantity of research on chemotherapy focused on finding better treatments that prolong overall survival, disease free survival and progression free survival. Quality of life is inconsistently reported in trials which address a primary outcome of overall survival. Therefore we have opted to transfer this outcome to the list of secondary outcomes to be considered in future updates of this review as appropriate. |
| Glenny[8] | Types of outcomes: The protocol for this review stated that quality of life would be a primary outcome for this review. Quality of life is an important outcome, for both patients with oral cavity and oropharyngeal cancers and their doctors. However, quality of life is infrequently and inconsistently reported in trials which address a primary outcome of overall survival. Therefore we have opted to transfer this outcome to the list of secondary outcomes to be considered in future updates of this review as appropriate. |
| Cheng[9] | We did not collect data on adverse events from interventions for erosive lichen planus by running separate searches looking specifically for adverse effects of treatments used. This was an over-ambitious goal and an under-estimation of the extensive search required to fulfil this. We added in a secondary outcome (j) reduction in target/mean lesion size (for oral lesions), which was measured by two studies |
| Ashley[10] | Adverse events were added as an outcome |

**References**

[1] Guo J, Li C, Zhang Q, Wu G, Deacon SA, Chen J, et al. Secondary bone grafting for alveolar cleft in children with cleft lip or cleft lip and palate. In: The Cochrane Collaboration, editor. Cochrane Database Syst. Rev., Chichester, UK: John Wiley & Sons, Ltd; 2011.

[2] Yengopal V, Harnekar SY, Patel N, Siegfried N. Dental fillings for the treatment of caries in the primary dentition. In: The Cochrane Collaboration, editor. Cochrane Database Syst. Rev., Chichester, UK: John Wiley & Sons, Ltd; 2009.

[3] Rasines Alcaraz MG, Veitz-Keenan A, Sahrmann P, Schmidlin PR, Davis D, Iheozor-Ejiofor Z. Direct composite resin fillings versus amalgam fillings for permanent or adult posterior teeth. In: The Cochrane Collaboration, editor. Cochrane Database Syst. Rev., Chichester, UK: John Wiley & Sons, Ltd; 2014.

[4] Coulthard P, Kushnerev E, Yates JM, Walsh T, Patel N, Bailey E, et al. Interventions for iatrogenic inferior alveolar and lingual nerve injury. In: The Cochrane Collaboration, editor. Cochrane Database Syst. Rev., Chichester, UK: John Wiley & Sons, Ltd; 2014.

[5] Daly B, Sharif MO, Newton T, Jones K, Worthington HV. Local interventions for the management of alveolar osteitis (dry socket). Cochrane Database Syst Rev 2012;12:CD006968. doi:10.1002/14651858.CD006968.pub2.

[6] Coulthard P, Bailey E, Esposito M, Furness S, Renton TF, Worthington HV. Surgical techniques for the removal of mandibular wisdom teeth. In: The Cochrane Collaboration, editor. Cochrane Database Syst. Rev., Chichester, UK: John Wiley & Sons, Ltd; 2014.

[7] Furness S, Glenny A-M, Worthington HV, Pavitt S, Oliver R, Clarkson JE, et al. Interventions for the treatment of oral cavity and oropharyngeal cancer: chemotherapy. Cochrane Database Syst Rev 2011:CD006386. doi:10.1002/14651858.CD006386.pub3.

[8] Glenny A-M, Furness S, Worthington HV, Conway DI, Oliver R, Clarkson JE, et al. Interventions for the treatment of oral cavity and oropharyngeal cancer: radiotherapy. Cochrane Database Syst Rev 2010:CD006387. doi:10.1002/14651858.CD006387.pub2.

[9] Cheng S, Kirtschig G, Cooper S, Thornhill M, Leonardi-Bee J, Murphy R, et al. Interventions for erosive lichen planus affecting mucosal sites. Cochrane Database Syst Rev 2012;2.

[10] Ashley PF, Parekh S, Moles DR, Anand P, Behbehani A. Preoperative analgesics for additional pain relief in children and adolescents having dental treatment. Cochrane Database Syst Rev 2012;9:CD008392. doi:10.1002/14651858.CD008392.pub2.
